# A new sensitive and accurate model to predict moderate to severe obstructive sleep apnea in patients with obesity

**DOI:** 10.1097/MD.0000000000016687

**Published:** 2019-08-09

**Authors:** Sofie Ahlin, Melania Manco, Simona Panunzi, Ornella Verrastro, Giulia Giannetti, Anna Prete, Caterina Guidone, Alessandro Di Marco Berardino, Luca Viglietta, Anna Ferravante, Geltrude Mingrone, Flaminio Mormile, Esmeralda Capristo

**Affiliations:** a Department of Molecular and Clinical Medicine, Sahlgrenska Academy, University of Gothenburg, Gothenburg, Sweden; b Department of Internal Medicine, Fondazione Policlinico Universitario A. Gemelli IRCCS, Università Cattolica del Sacro Cuore Rome; c Research Unit for Multifactorial Diseases, Obesity and Diabetes Scientific Directorate, Bambino Gesù Children's Hospital, IRCCS; d CNR-IASI, Istituto di Analisi dei Sistemi ed Informatica “A.Ruberti”, BioMatlab; e Department of Respiratory Diseases, Fondazione Policlinico Universitario A. Gemelli IRCCS, Università Cattolica del Sacro Cuore Rome, Italy; f Division of Diabetes & Nutritional Sciences, Faculty of Life Sciences & Medicine, King's College London, London, United Kingdom.

**Keywords:** obesity, prediction risk chart, sleep apnea

## Abstract

Obstructive sleep apnea (OSA) has a high prevalence in patients with obesity. Only patients with clinical symptoms of OSA are admitted to polysomnography; however, many patients with OSA are asymptomatic. We aimed to create and validate a population-based risk score that predicts the severity of OSA in patients with obesity.

We here report the cross-sectional analysis at baseline of an ongoing study investigating the long-term effect of bariatric surgery on OSA. One-hundred sixty-one patients of the Obesity Center of the Catholic University Hospital in Rome, Italy were included in the study. The patients underwent overnight cardiorespiratory monitoring, blood chemistry analyses, hepatic ultrasound, and anthropometric measurements. The patients were divided into 2 groups according OSA severity assessed by the apnea-hypopnea index (AHI): AHI < 15 = no or mild and AHI ≥ 15 moderate to severe OSA. A statistical prediction model was created and validated. C statistics was used to evaluate the discrimination performance of the model.

The prevalence of OSA was 96.3% with 74.5% of the subjects having moderate/severe OSA. Sex, body mass index, diabetes, and age were included in the final prediction model that had excellent discrimination ability (C statistics equals to 83%). An OSA risk chart score for clinical use was created.

Patients with severe obesity are at a very high risk for moderate or severe OSA in particular if they are men, older, more obese, and/or with type 2 diabetes. The OSA risk chart can be useful for general practitioners and patients as well as for bariatric surgeons to select patients with high risk of moderate to severe OSA for further polysomnography.

## 1 Introduction

In the general population, obstructive sleep apnea has a prevalence of approximately 10–17% in men and 3–9% in women.^[1]^ Obesity represents the major risk factor for OSA development with 58% of moderate to severe OSA being associated with obesity.^[2–4]^

Patients with OSA are at high risk of all-cause mortality,^[5,6]^ particularly cardiovascular related,^[7]^ and have a 6 times higher risk of being involved in a traffic accident.^[8]^ In addition, OSA is linked to nonalcoholic fatty liver disease^[9]^ and the intermittent hypoxia during sleep that arises during OSA may lead to oxidative stress, lipid peroxidation, and progress of the disease.^[10]^ Furthermore, OSA affects the quality of life^[11]^ which, at least partly, can be improved by treatment.^[12]^ OSA is also important in a perioperative setting as it is a risk factor of postoperative complications^[13]^ and the identification and treatment of OSA is recommended before bariatric surgery.^[14]^ Hence, identifying patients with OSA in the general population as well as in a perioperative setting is essential. However, more than 80% of the patients with OSA remain undiagnosed.^[15]^

Screening questionnaires might help to identify patients with OSA. Nevertheless, the sensitivity, specificity, and predictive value of self- or interview-administered questionnaires depend from many variables including the specific reference population, the OSA prevalence in the study sample and the disease severity.^[16,17]^ Even more importantly, the patients often underreport their symptoms or are asymptomatic which makes it harder to detect the disease.^[18]^

Standard laboratory polysomnography is considered the golden standard for the diagnosis of OSA, although portable sleep monitoring systems at home (PM) are gaining more and more interest in relation to their cost-effectiveness. The cost for PM has been calculated to be 256.5 ± 16.9 EUR or 348.2 ± 22.9 USD which can be compared with the laboratory polysomnography cost of 548.1 ± 45.7 EUR or 744.1 ± 62.0 USD.^[19]^ A recent study evaluated in the general population the effectiveness to assess OSA with home sleep testing without a sleep medicine specialist. The results were promising where the diagnosis was missed in <6% of the patients and 16.8% of the patients were misclassified.^[20]^ However, there is still a need to identify those patients who need to undergo polysomnography.

Here we aim to report the prevalence of OSA in a high risk population of patients evaluated for bariatric surgery, to highlight those factors associated with its severity and to create and validate a model to predict moderate to severe OSA in these patients.

## 2 Methods

### 2.1 Ethics statement

This clinical study (ClinicalTrial.gov ID NCT03223467) has been approved by the ethic committee of the Catholic University of the Sacred Heart in Rome, Italy. All participants gave informed consent to participate in the study.

### 2.2 Study design

We here report the cross-sectional analysis at baseline of an ongoing study investigating the long-term effect of bariatric surgery on OSA. The objective for the present study was to investigate the prevalence and severity of OSA in patients evaluated for bariatric surgery, to study the possible links between components in the metabolic syndrome and the severity of OSA and to create and validate a model that predicts moderate to severe OSA.

One-hundred sixty-one patients screened for bariatric surgery were enrolled between January 1, 2012 and December 31, 2012 and between January 1, 2017 and December 31, 2017. Initially, 86 patients were recruited in 2012 and 85 patients were recruited during 2017. Nine patients (4 patients during 2012 and 5 patients during 2017) were excluded from the study because they were not able to perform polysomnography. The patients were recruited during their hospitalized evaluation for bariatric surgery at the Obesity Center at Catholic University Hospital in Rome, Italy. The patients underwent usual evaluation for possible bariatric surgery with fasting blood samples, anthropometric measurements, and ultrasound of the abdomen to evaluate hepatic steatosis. An overnight cardiorespiratory monitoring with VitalNight 8 (VitalAire, Milan, Italy) was also performed to investigate the presence and severity of OSA. All blood samples were analyzed at the central laboratory of the Catholic University Hospital.

The participants underwent a 3-hour oral glucose tolerance test (OGTT). Patients who had a plasma glucose value above 200 mg/dL after 2-hour OGTT or already had a known diagnosis of type 2 diabetes (T2D) were categorized as patients with T2D. Presence of antihypertensive medications was considered as a diagnosis of hypertension.

### 2.3 Statistical analysis

Patients were classified into 2 groups according to the OSA severity on the basis of their apnea-hypopnea index (AHI): AHI < 15 was classified as no or mild OSA, whereas AHI ≥ 15 was classified as moderate or severe OSA. Student *t* test was used to evaluate possible differences between the 2 groups for continuous variables and Fisher exact test was used to study possible association between study groups and categorical variables. All statistical analyses were performed with IBM SPSS Statistics for Windows (version 21.0; IBM Corp, Armonk, NY) or the R package.

#### 2.3.1 OSA prediction model

The AHI score was considered as a binary variable with class 0 for no or mild OSA (AHI <15) and class 1 for moderate to severe OSA (AHI values ≥15). Nine variables for the prediction of the binary AHI outcome were evaluated on the study population of 161 patients with the aim of finding the best set of predictors. The variables initially included in the model were: age, sex, T2D (0 = absence, 1 = presence), fasting glucose, body mass index (BMI), hypertension medication (0 = no administered drug, 1 = administered drug), aspartate transaminase (AST), alanine transaminase (ALT), and hepatic steatosis. The validity of the full model (the model including all the nine measured predictors) was assessed by a model validation approach (see Section 2.3.2 for more details). On the basis of the obtained results, a reduced model was then tested and validated.

#### 2.3.2 Model validation

To establish the best model for predicting the probability of moderate or severe OSA occurrence, the following procedure was set up.

The data set was split into a fitting population and a validation population by using the internal/validation/data-splitting method. The internal/validation/data-splitting method was chosen to limit the possible increase in bias that can occur with other techniques for internal validation such as Bootstrapping technique if the original study population contains biases. A random portion of the population (70%, n = 113) was used as fitting population for model development. The number of subjects in the fitting population was based on the rule-of-thumb that N = 104 + m (m = the number of predictors) when testing for partial correlations.^[21]^ The model was then tested, in terms of its performance on the remaining validation population (30%, n = 48). Computation of the C statistic was used to indicate the discrimination performance of the model. The C statistic represents a measure of the model's performance in terms of the model discrimination ability to separate subjects with different outcomes, and for the logistic model, it is equivalent to the area under the curve of the receiver operating characteristic (ROC) curve associated to the model.^[22]^ Values of C between 0.8 and 0.9 were considered to give an excellent discrimination. An OSA risk chart was constructed based the probability of moderate to severe OSA.

The steps of the procedure are summarized as follows:

1. Data splitting: The studied population was split into a fitting population and a validation population.
2. Model fitting: The model was fitted onto data from the fitting population and the model coefficients for the variables included into the model, along with their standard errors and significance levels, were computed.
3. The prediction model was applied to the validation population.
4. Computation of the probabilities: For each subject, both from the fitting population and the validation population, the probability of the studied outcome was computed on the basis of the patient's covariate values. Each subject was therefore characterized by the values of the covariate set, the outcome (AHI 0/1) and by the estimated probability of the outcome.
5. Computation of the C statistics: For both the fitting and the validation populations, the C statistics was computed. The accuracy (percentage of true positive and negative on the total subjects) of the prediction model was also computed.
6. Procedure repetition: The entire procedure (data splitting, model fitting, probability computation) was repeated 200 times.
7. Model fitting onto the whole population: The model was then fitted using all the original data.
8. Results analysis: At the end of the procedure, the distribution of different indicators was computed: for each variables, frequency histograms of the estimated coefficients along with their associated standard errors and levels of significance were plotted. The importance index of each variable computed from each data splitting and model fitting was also plotted.

The estimated coefficients from the whole population were compared with the respective distributions (they should be centered around the estimates obtained on the whole sample); the distribution of the variability of the model coefficients indicates to which extent the results are dependent from the subpopulation used; comparison between the distribution of the C statistic computed on the fitting and the validation sample, along with the distribution of their Delta provide indication of the discrimination power of the model. It is expected that the C statistic is larger for the fitting sample than for the validation sample. A model with a high discrimination power presents small drops in the values of the C statistic when computed on the validation sample with respect to values computed on the fitting sample for which the values are expected to be larger (C Delta distribution centered around the zero).

On the basis of the obtained results on the full model, the entire procedure is repeated on the subset of the most important variables to test and validate the final reduced model including the most meaningful predictors. Once the model validation is assessed the fitted coefficients used to compute the probability of the outcome on a generic patient will be those obtained from the “reduced model” fitting on the whole population sample.

## 3 Results

### 3.1 Patient characteristics

Patient characteristics are reported in Table 1. The proportions of men and women were balanced in the whole study population (49.7% vs 50.3%). A very high prevalence of OSA, equal to 96.2% of the patients, was observed. The majority, about 74.5%, of the patients suffered from OSA that was considered to be either moderate (21.7%) or severe (52.8%), whereas 21.7% had mild OSA.

**Table 1:**
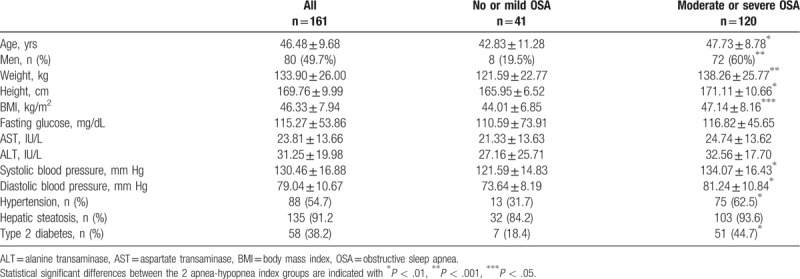
Baseline characteristics of study participants.

The proportions of men and women in the 2 AHI groups were different. Only 19.5% of the patients with no or mild OSA were men compared to 60% of the patients with moderate or severe OSA. BMI was higher in patients with moderate/severe OSA than in patients with no/mild OSA (47.14 ± 8.16 vs 44.01 ± 6.85 kg/m^2^, *P* < .05). More patients with moderate/severe OSA had hypertension (62.5% vs 31.7%, *P* < .01) and T2D (44.7% vs 18.4%, *P* < .01) than those with no/mild OSA. Hepatic steatosis assessed by ultrasonography was similar in the 2 categories.

### 3.2 Selection of variables and validation of a prediction model of OSA

Four of the 9 variables included in the full model were entered the final reduced model. All the selected variables from the full model, age, sex, BMI, and diabetes status, displayed a median importance coefficient >50%, a median *P*-value <.15, and the average of the coefficient distributions were centered around the estimated coefficient obtained by fitting the model on the whole study population. Estimated coefficients from the whole sample, the average of the coefficient distributions and the median of the *P*-values and importance index of the respective distributions from the full model are displayed in Table 2.

**Table 2:**
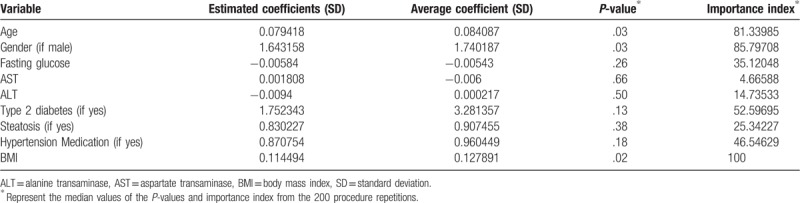
Descriptive statistics from the fitting distributions for the “full model” along with the estimated coefficients from the whole sample.

**Figure 1:**
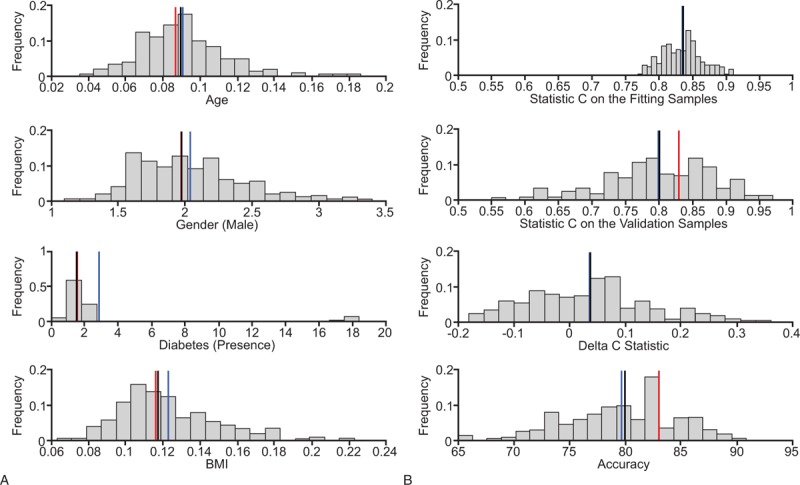
(A) Distributions of the coefficient estimates along with the average value (blue vertical line), median value (vertical black line), and the coefficient estimates on the whole sample (vertical red line) in the “reduced model.” (B) Distribution of the C statistics computed over the fitting samples and over the validation samples along with the distribution of the absolute deltas and of the accuracy. Blue lines and black lines represent the average and the median values of the distributions respectively whereas the red lines represent the obtained values from the model fitting over the whole sample.

Coefficient estimates of the variables in the final reduced model in the training sample were similar to the estimated coefficients when the model was fitted onto the whole population (Table 3). Distributions of the coefficient estimates for age, sex, T2D status, and BMI in the training sample are shown in Figure 1A, where the average and median values of the distributions are indicated together with the obtained values from the model fitting over the whole study population.

**Table 3:**
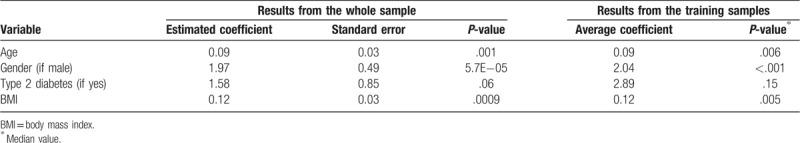
Descriptive statistics from the fitting distributions for the “reduced model” along with the estimated coefficients from the whole sample.

### 3.3 Discrimination power of the model

C statistics were computed to indicate the discrimination power of the model (Fig. 1B). As expected, the average of C statistics was a little higher in the fitting sample than in the validation samples (83 ± 3% and 80 ± 8%, respectively) with the absolute delta of 3.6 ± 11% and the distribution of the accuracy of 80 ± 5% for the reduced model. When the reduced model was applied on the whole study population, C statistics was 83%, indicating an excellent discrimination of the reduced model in separating subjects with and without moderate/severe OSA.

**Figure 2:**
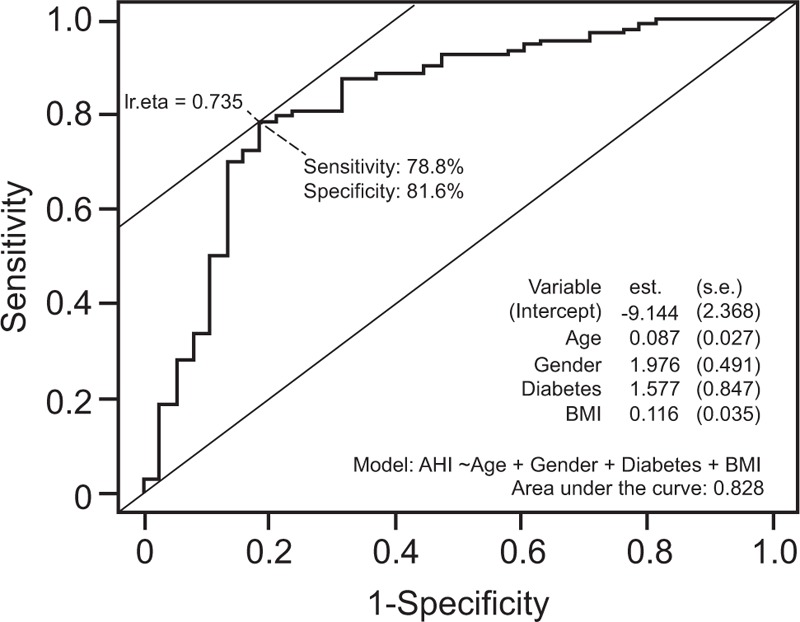
Receiver operating characteristic curve reporting the sensitivity and specificity of the reduced model of obstructive sleep apnea severity. BMI = body mass index.

An ROC curve was constructed and an optimal cut-off value of 0.735 for the model was calculated (Fig. 2). At this value, the final reduced model displayed a sensitivity of 78.8% and a specificity of 81.6% to identify patients with moderate or severe OSA.

### 3.4 OSA risk score for clinical use

**Figure 3:**
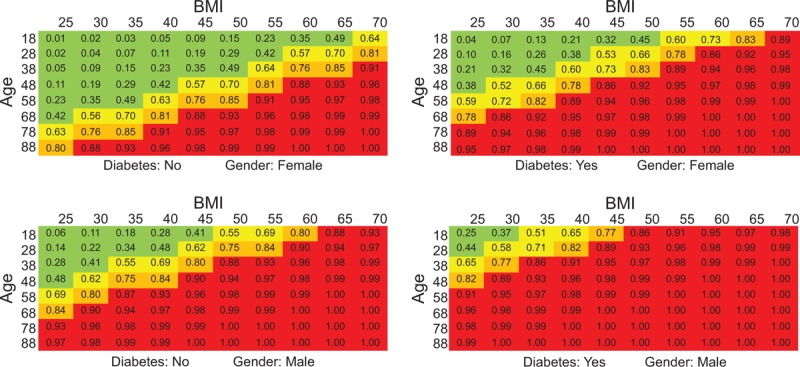
Obstructive sleep apnea risk charts. Very low risk is in green, moderate risk in yellow, high risk in orange, and very high risk in red. Different charts are reported for absence or presence of type 2 diabetes and for gender. Body mass index (BMI) increases by 5 kg/m^2^ while age increases by 10 years.

The final reduced model was used to create an OSA risk chart score. The cut-off values obtained from the ROC curve was used to identify patients at risk. Patients presenting probability of developing moderate/severe OSA <0.5 were classified at very low risk (green), estimated probabilities between 0.5 and 0.735 were associated to moderate risk (yellow), values between 0.735 and 0.85 were associated to high risk (orange) and patients presenting probabilities >0.85 were classified at very high risk. The chart is reported in Figure 3. In the OSA risk chart score, 4 different charts are used depending on the categorical variables in the model (sex and T2D status) and color code indicate risk of moderate/severe OSA.

## 4 Discussion

In this cross-sectional study in patients evaluated for bariatric procedures, the prevalence of OSA was very high. Several variables were associated with OSA severity but the best predictors of moderate to severe OSA were age, sex, BMI, and diabetes status, where men, older, higher BMI, and with T2D being at progressively higher risk for OSA of moderate or severe degree. Based on our prediction model, we also constructed an OSA risk chart that can be helpful for clinicians to select patients for admission to polysomnography.

In our study, we 1st evaluated how known risk factors for OSA could predict the severity of the disease and selected the best predictors for our final model. To obtain a model that could fit a general population with obesity a validation approach was implemented in the statistical analyses. The statistical methods used in this study give the possibility to evaluate if the model is able to describe the dependence of the outcome on the selected predictors both in the analyzed sample and in the entire reference population. By performing a data-splitting method respecting the minimal numbers of subjects for multiple regression analysis and maintaining the proportions of subjects in each AHI group, we have tried to limit possible sources of bias that could have occurred using other techniques for internal validation.

Our prediction model displayed that increasing age and BMI together with presence of T2D and male sex were identified as risk factors for moderate or severe OSA in our study. These results are in line with previous reported risk factors for OSA^[23]^ and further strengthen previous studies where links between OSA and altered glucose homeostasis have been reported.^[24–29]^

Based on our prediction model, we have created an OSA risk chart that can be useful for general practitioners as well as for bariatric surgeons when selecting patients for admission to polysomnography. The C statistic showed an excelled discrimination (83%) between patients with low to high risk and a cut-off of 0.735 provided a sensitivity of 78.8% and a specificity of 81.5%. According to the American Society for Metabolic and Bariatric Surgery, ASMBS, the recent clinical practice only admits patients with clinical symptoms of OSA to polysomnography^[30]^ and many of the questionnaires used for screening of OSA focus on symptoms. However, many patients with OSA are asymptomatic.^[18]^ Our OSA risk chart is based on anthropometry and blood chemistry and can be used as an important complement to the questionnaires to select patients for admission to polysomnography.

About 96% of our study population had OSA and about 75% were considered to have OSA of moderate or severe degree. The prevalence of OSA in the bariatric surgery population has been previously reported in the range 71% to 91% which means that our prevalence is in the upper level^[18,31–33]^ probably depending on differences in study population composition. Our high prevalence number of OSA highlights the importance of screening for OSA. In addition, patient eligible for bariatric surgery but not referred to bariatric surgery or obesity clinics, where evaluation of OSA is common, could be at high risk of not having a diagnosis and thus they would not receive a correct treatment and the treatment benefits. However, selecting the right patients for polysomnography is important due to limited health care budget. In our study population, 25% of the subjects had no OSA or mild OSA. Screening with our OSA risk chart would have resulted in savings ranging from 7225.15 USD (83% of 25 = 20.75 *×* USD 348.2) to USD 15400.1 (83% of 25 = 20.75 *×* USD 744.1) for portable sleep monitoring systems use at home or polysomnography test at the hospital, respectively.

In conclusion, our study shows that morbidly obese subjects are at a very high risk for moderate to severe OSA in particular if they are men, older, have higher BMI and with T2D. All predictors of the severity of OSA in our study are well-known risk factors for OSA but to help clinicians to identify patients with high risk of moderate and severe OSA, we created an OSA risk chart score that can predict the presence of OSA of moderate to severe intensity with 83% accuracy. Our OSA risk chart is not dependent on the symptoms of OSA which many other OSA screening tools are, such as the STOP-Bang questionnaire and Berlin questionnaire.^[34]^ Hence, our OSA risk chart can be especially useful to identify patients that do not report symptoms even though they still have the disease.^[18]^ However, external validation in a different population is needed to further evaluate our OSA risk chart. We hope that our OSA risk chart in the future can be useful for general practitioners and patients as well as for bariatric surgeons and can permit to address to polysomnography only those patients with moderate or severe OSA with a particular focus to those patients who do not report symptoms of OSA but still have the disease.

## Author contributions

**Data curation:** Sofie Ahlin, Ornella Verrastro, Giulia Giannetti, Anna Prete, Caterina Guidone, Alessandro Di Marco Berardino, Luca Viglietta, Anna Ferravante, Flaminio Mormile.

**Formal analysis:** Sofie Ahlin, Simona Panunzi.

**Investigation:** Geltrude Mingrone, Esmeralda Capristo.

**Methodology:** Simona Panunzi, Geltrude Mingrone, Flaminio Mormile, Esmeralda Capristo.

**Project administration:** Ornella Verrastro, Giulia Giannetti, Anna Prete, Geltrude Mingrone, Esmeralda Capristo.

**Resources:** Melania Manco.

**Supervision:** Melania Manco, Geltrude Mingrone, Flaminio Mormile, Esmeralda Capristo.

**Writing – original draft:** Sofie Ahlin, Melania Manco, Geltrude Mingrone, Esmeralda Capristo.

**Writing – review & editing:** Sofie Ahlin, Melania Manco, Simona Panunzi, Ornella Verrastro, Giulia Giannetti, Anna Prete, Caterina Guidone, Alessandro Di Marco Berardino, Luca Viglietta, Anna Ferravante, Geltrude Mingrone, Flaminio Mormile, Esmeralda Capristo.

Sofie Ahlin orcid: 0000-0003-0619-2683.
